# Redox-Sensitive Gelatin/Silica-Aptamer Nanogels for Targeted siRNA Delivery

**DOI:** 10.1186/s11671-019-3101-0

**Published:** 2019-08-14

**Authors:** Xueqin Zhao, Yinyin Xi, Yongming Zhang, Qiuyan Wu, Ruiyuan Meng, Bin Zheng, Lei Rei

**Affiliations:** 1 College of Life Science and Medicine, Zhejiang Sci-Tech University, Hangzhou, 310018 People’s Republic of China; 2 Department of Otolaryngology, Zhejiang Provincial People’s Hospital, People’s Hospital of Hangzhou Medical College, Hangzhou, 310014 People’s Republic of China; 3 Department of Biomaterials, College of Materials, Xiamen University, Xiamen, 361005 People’s Republic of China

**Keywords:** siRNA delivery, Anti-nucleolin aptamer AS1411, Nucleolin targeting, Gene therapy, RNA interference

## Abstract

RNA interference (RNAi) has potential advantages over other gene therapy approaches due to its high specificity and the ability to inhibit target gene expression. However, the stability and tissue-specific delivery of siRNA remain as the biggest obstacles for RNAi therapeutics. Here, we developed such a system by conjugating gelatin-based nanogels with the nucleolin-targeted AS1411 aptamer and deoxynucleotide-substituted siRNA together (Apt-GS/siRNA) via a disulfide linker to achieve transient docking of siRNA. These Apt-GS/siRNA nanogels demonstrated favorable release of siRNA under reducing conditions owing to disulfide cleavage. Furthermore, this smart system could electively release siRNA into the cytosol in nucleolin-positive cells (A549) by a glutathione-triggered disassembly and subsequently efficient RNAi for luciferase. Besides, disulfide-equipped Apt-GS nanogels showed good biocompatibility in vitro. Taken together, this redox-responsive, tumor-targeting smart nanogels display great potential in exploiting functionalized siRNA delivery and tumor therapy.

## Introduction

RNA interference (RNAi) is a sequence-specific silencing of genes and also currently being evaluated as a most promising method in the treatment of a wide range of diseases including genetic disorders, cancers, and infectious diseases, owing to its high specificity and low toxicity [1]. Small interfering RNAs (siRNAs) with double-stranded RNA molecules are more resistant to nuclease degradation than antisense oligonucleotides and therefore show advantages over antisense therapy. Incorporation of chemically modified nucleotides into siRNAs was used to increase or decrease the efficacy and the duration of RNAi. Replacing the ribonucleotides of sense strand with deoxynucleotides has been proved could increase the stability of sense strand and keep ∼ 40% efficacy of RNAi [2, 3]. However, tissue-specific delivery of siRNA remains to be a big obstacle for its applications despite that the great gene knockdown potencies observed in the in vitro studies [4, 5].

Nanoparticle-based delivery has potential advantages in effective siRNA stabilization and further modification for site-specific delivery [6–8]. Productive site-directed delivery of siRNA can enhance transfection and reduce off-target effects, which are required by most practical applications [9]. Nucleolin is associated with diverse biological processes and wildly expressed in the nucleus and cytoplasm of various normal cells. Moreover, nucleolin is highly expressed on the plasma membranes of actively proliferating cancer cells relative to their normal counterparts and hence used as an attractive target for antineoplastic treatments. Aptamer AS1411 (also known as AGRO100), a G-quartet DNA aptamer, can strongly bind to cell surface nucleolin and block the antiapoptotic pathway in cancer cells by combining with nuclear factor-kB essential modulator, which has reach phase II clinical trials as the first nucleic acid aptamer drug for the treatment of cancer in humans [10, 11]. Thus far, AS1411 has been successfully conjugated to various nanoparticles and internalized them into cancer cells [12–15]. Gelatin has been employed for siRNA encapsulation due to its excellent biocompatibility, biodegradability, and gelation properties [16–18]. Our group has explored a series of siloxane-crosslinked gelatin nanogels (GS NGs) with controlled size and surface charge, demonstrating the transfection efficiency of GS NGs in vitro and in vivo [19, 20].

Besides intracellular barriers, intracellular barriers after internalization including efficient disassembly of siRNA and endosomal escape equally remain challenging. Conjugation of siRNA to a carrier through labile or nonlabile bonds is a promising method for overcoming this delivery challenge. Stimuli-responsive release under acidic pH and redox potential has received great interest. Disulfide cross-link displays great potential in a burst of drug release via cleaved linkages in tumor cells due to higher glutathione (GSH) level in the extracellular environment [21, 22]. It has reported that poly (lactic-co-glycolic acid) (PLGA)/siRNA conjugates formed via a disulfide bond exhibit an enhanced encapsulation and delivery efficiency [23].

**Scheme 1:**
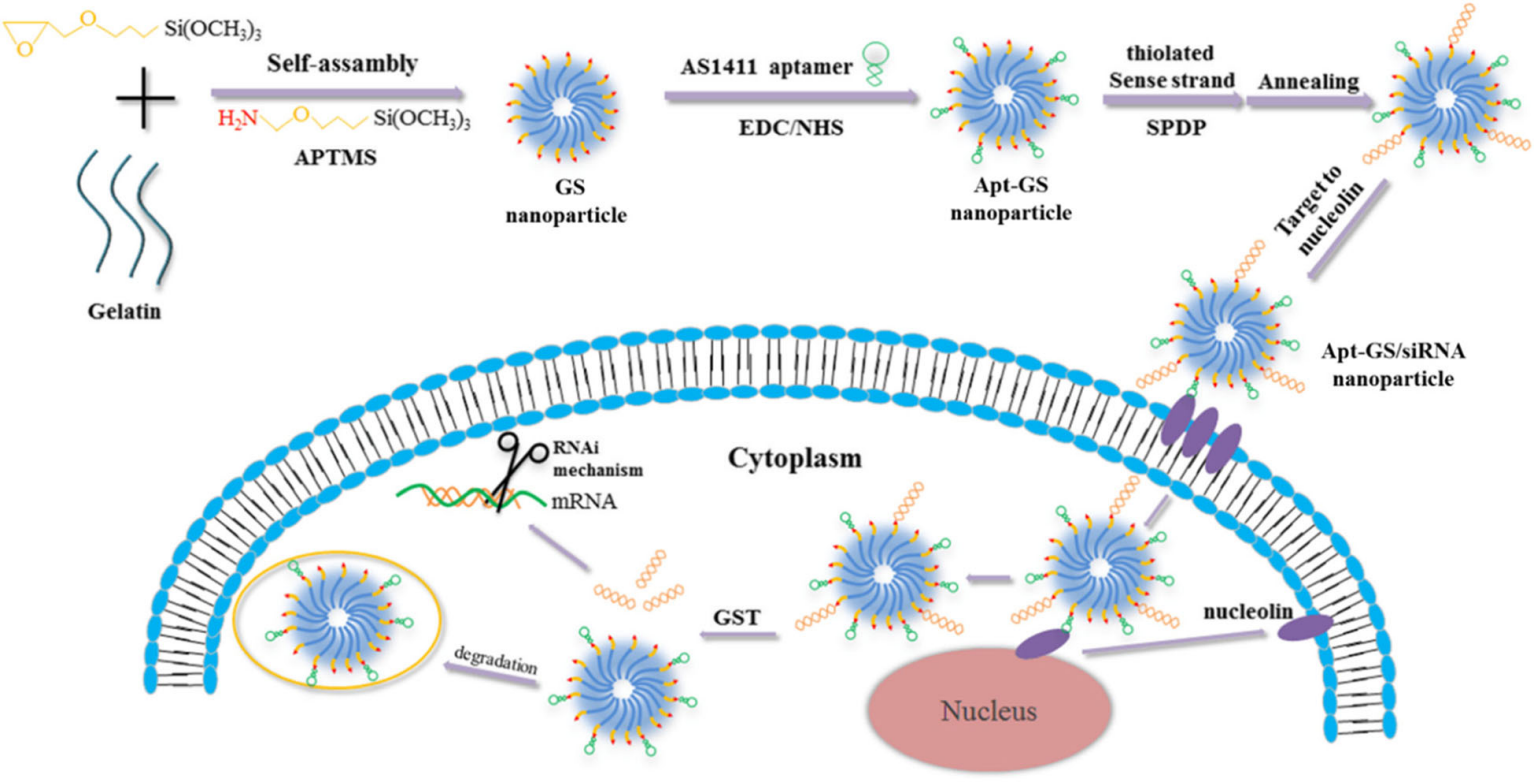
A redox-responsive and tumor-targeting Apt-GS NGs was developed for siRNA delivery with disulfide conjugation and aptamer AS1411

In this work, hybrid nanogels based on GS with dual functions of redox responsiveness of disulfide conjugation and specific tumor targeting of aptamer AS1411 was explored as a siRNA carrier for cancer therapy (Scheme 1). We aim to investigate whether AS1411 functional GS NGs would improve effective cellular internalization and achieve tumor-specific gene silencing of luciferase model gene in vitro. Besides, the safety of this system was also evaluated in vitro.

## Methods

### Materials

Gelatin (bloom number: 240-270, pH 4.5–5.5) was purchased from BBI Company Inc. (USA). *N*-succinimidyl 3-(2-pyridyldithio) propionate (SPDP), 3-glycidoxypropyl-trimethoxysilane (GPSM), and 3-aminopropyl-trimethoxysilane (APTMS) were purchased from Sigma-Aldrich Co. (USA). 3-(4, 5-Dimethylthiazolyl-2)-2, 5-diphenyltetrazolium bromide (MTT) was purchased from Amresco Co. (USA). Dulbecco’s modified Eagle’s medium (DMEM), fetal bovine serum, penicillin, and streptomycin were obtained from Hyclone Co. (USA). AS1411 aptamer, 5′-FAM-labeled AS1411, and negative control DNA (TDO) were synthesized by Sangon Biotech Co. (China). Thiol sense strand of the Luc-siRNA, 5′-SH-(CH_2_)_6_-CTTACGCTGAGTACTTCGATT-3′ (deoxynucleotides substitute for ribonucleotides) and anti-sense strand, 3′-TTGAAUGCGACUCAUGAAGCU-5′, and FAM-labeled anti-sense strand at the 3′ end were synthesized and purified with HPLC by Gene Pharma Co. (China). Lipofectamine 2000, luciferase plasmid pGL3, and luciferase assay system were purchased from Promega Co. (USA). The malignant human lung adenocarcinoma A549 cells and normal NIH 3T3 fibroblasts were used and provided by the Biomedical Engineering Centre of Xiamen University (China). All materials used were of analytical grade and without further purification. The glassware was thoroughly cleaned and rinsed with deionized water.

DNA sequences are as follows:

AS1411 aptamer: 5′-GGTGGTGGTGGTTGTGGTGGTGGTGGTTTTTTTTTTTT-3′, Control TDO: 5′-CACCGGGAGGATAGTTCGGTGGCTGTTTTTTTTTTTTT-3′

#### Preparation and Characterization of Apt-GS/siRNA Complexes

Amino-functionalized gelatin/silica nanogels (GS NGs) were first prepared by a sol-gel procedure, as previously described [19]. Typically, 0.2 g of GPSM was added to 20 mL of 0.75% gelatin solution in HCl solution (pH = 3) at 60 °C under 30 min of stirring, and subsequently adding 0.08 g APTMS and incubating for another 24 h. The obtained GS NGs were purified by centrifugation (14,000 rpm, 25 °C, 12 min) for three times. Secondly, GS NGs (0.5 mL, 5 g/L in PBS) were treated with 25 uL SPDP (20 mM in DMSO) for 60 min at room temperature, followed by the addition of thiolated AS1411 (1 mg/mL) and stirring for 12 h. Apt-GS nanogels were obtained by centrifugation (14,000 rpm, 25 °C, 12 min) and purified with deionized water thrice. Thirdly, the obtained SPDP-activated Apt-GS NG suspension (80 mL, 5 mg/mL) was directly mixed with siRNA sense strand overnight at 4 °C. After purification by centrifugation, the siRNA anti-sense strand was added and incubated for 5 min at 94 °C, then annealed for 20 min at 47 °C to form Apt-GS/siRNA complexes.

The surface morphologies of AS1411-GS and AS1411-GS/siRNA complexes were examined by transmission electron microscopy (TEM) (Hitachi S-4300, Japan). The particle size and zeta potentials of each sample were measured by a Nano-ZS Zetasizer dynamic light scattering detector (Malvern Instruments, UK). The effective particle diameters were calculated from the autocorrelation function using the Malvern Zetasizer software assuming a log-normal distribution. The conjugations of GS and AS1411 or siRNA were determined by collecting and measuring the concentration of unattached FAM-labeled DNA using F-7000 fluorescence microscopy (Olympus-IX73, Japan). Total amino group (-NH_2_) levels on the surface of GS nanogels were quantitatively determined by using the ninhydrin colorimetric reaction. The amount was about 0.642 mmol/g.

#### Gel Electrophoresis Analysis

Gel electrophoresis was conducted at 100 V for 20–30 min using 2% (w/v) agarose gel in TBE buffer. Images were observed by irradiation with a gel documentation system (Tanon GIS-2008, China). In the Apt-GS/siRNA encapsulation assay, the naked siRNA, GS/siRNA, and Apt-GS/siRNA complexes were loaded into the gel without any additives. In redox-responsive assays, GS/siRNA and Apt-GS/siRNA complex were incubated with 10 mM GSH solution in PBS for 2 h before measurement.

#### In Vitro Cytotoxicity

The cytotoxicity against human lung adenocarcinoma A549 cells was evaluated by MTT assay. Briefly, A549 cells (1 × 10^4^ cells/well) were seeded in polystyrene 96-well culture plates and incubated for 24 h till the 70% confluence. After removing the culture medium, 100 μL of serum-free DMEM medium containing nanogels (100–600 mg/mL) was added to each well. Cells treated with medium only served as a negative control group. After 24 h co-incubation, 100 μL of fresh medium with 20 μL of MTT solution (5 mg/mL in PBS buffer) was added and cultured for another 4 h. Then, MTT solution was removed and 100 μL of dimethyl sulfoxide (DMSO) was added. After oscillating for 30 min in the dark, the absorbance of each well was measured by a micro-plate reader (TECAN DNA export, Swiss) at the 490 nm wavelength. All experiments were conducted in triplicate. The relative cell viability (%) was expressed as a percentage relative to the untreated control cells.

#### Cell Internalization

The cells are co-cultured with 25 μL of FAM-labeled Apt-GS and TDO-GS nanogels (2 mg/mL) for 10 h in serum-free medium at 37 °C. After washing three times with PBS, cells were fixed with paraformaldehyde (4% in PBS) for 30 min and then observed with confocal laser scanning microscope (Leica, Germany). To quantify the cellular uptake, the cells were treated with 25 μL of FAM-labeled AS1411-GS and TDO-GS (2 mg/mL) for a period (0.5–16 h) in serum-free medium at 37 °C. To evaluate the role of nucleolin in the cellular uptake of the Apt-GS NGs, the mixture of the prepared Apt-GS NGs and free AS1411 aptamers of varied concentrations (0.5, 5, and 50 μM) was incubated with the A549 cells for 8 h. Then, cells were re-suspended in ice-cold PBS, and the fluorescent cells with FAM-labeled nanogels were counted from 10,000 cells by using an EPICS XL flow cytometer (Beckman Coulter, USA). Data analysis was performed with EPICS XL flow cytometer software, and analytical gates were chosen as 1% of control cells falling within the positive region.

#### Gene Silencing

The ability to silence genes was examined using the combination of siRNA for luciferase and the luciferase-expressing cells. Briefly, luciferase-expressing A549 (2 × 10^5^ cells/well) and NIH 3 T3 fibroblasts (4 × 10^5^ cells/well) were seeded in 12-well plate and cultured overnight and then treated with 100 μL of test complexes (80 μg/mL) containing LUC-siRNA (or control siRNA) for 8 h. The samples were divided into three groups: (a) only Apt-GS/siRNA complexes for 8 h, (b) Apt-GS/siRNA complexes for 8 h followed by liposomes for further 1 h (named as A-GS/si→Lip group), and (c) co-incubation of Apt-GS/siRNA complexes and liposome for 8 h (A-GS/si+Lip group). The cells without any treatment and those transfected with luciferase plasmid pGL3 (Madison, WI, USA) were as controls. After further 40 h incubation, gene knockdown efficiency was investigated by quantifying the luciferase expression in cells using a luciferase assay system according to the manufacturer’s protocol. Experiments were carried out in triplicate, and data are shown as means ± standard errors of the means.

## Results and Discussions

### Synthesis and Characterization of GS-AS1411/siRNA Complexes

**Fig. 1:**
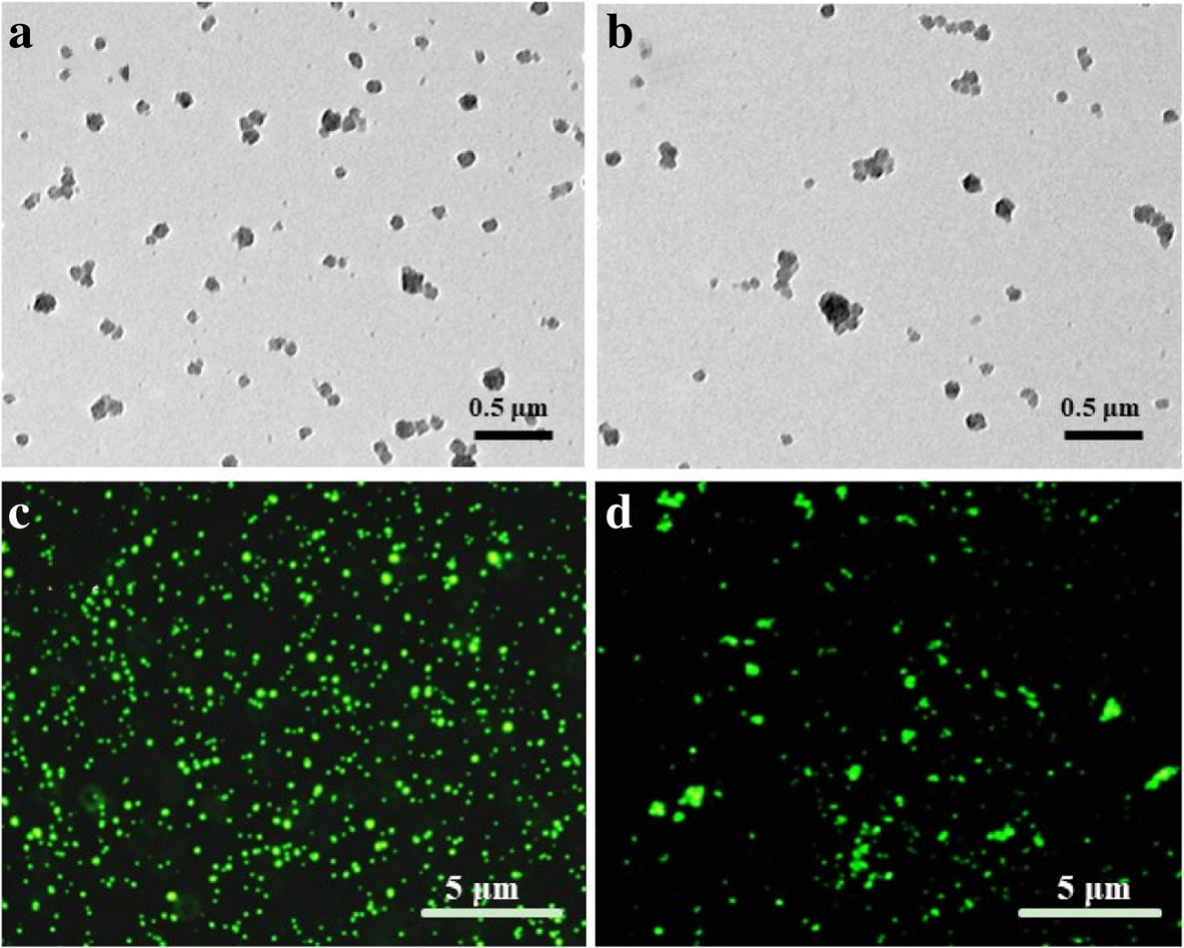
Images of Apt-GS (**a**, **c**) and Apt-GS/siRNA nanogels (**b**, **d**). **a**, **b** TEM images. Scale bars represent 0.5 μm. **c**, **d** Fluorescent images. Scale bars represent 5 μm

**Fig. 2:**
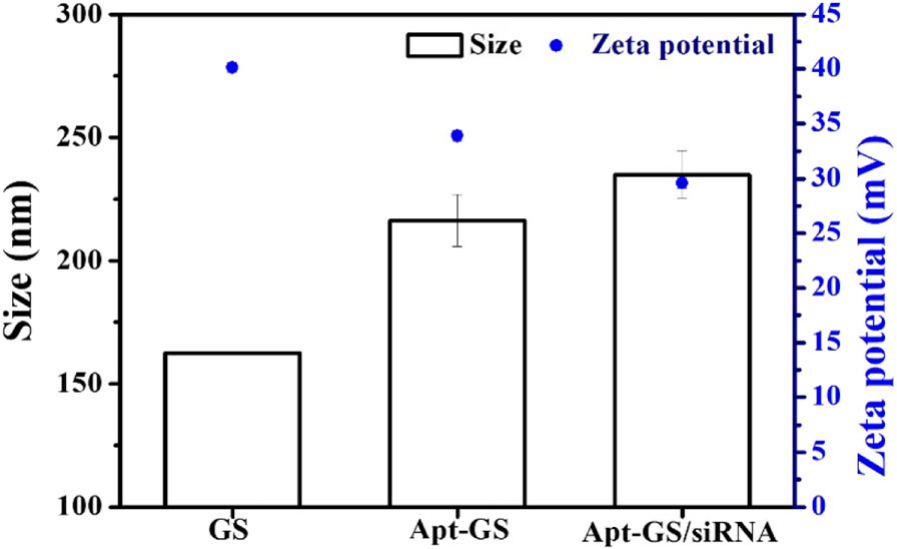
Hydrodynamic size and zeta potentials of GS, Apt-GS, and Apt-GS/siRNA nanogels

To construct the cancer-targeting siRNA carrier, gelatin/silica-AS1411 (Apt-GS) nanogel was designed (Scheme 1) using a sol-gel method [19, 20]. Typical TEM image (Fig. 1) revealed that blank GS and Apt-GS were dispersed spherical structures with average diameters of 170–200 nm. FAM-labeled DNA was used for the synthesis to confirm coupling of the AS1411 and siRNA onto GS NGs. The amount of conjugated aptamer on GS was approximately 0.65 μmol/g, as quantified using fluorescence spectra. Meanwhile, intense green fluorescence can be easily observed in the fluorescence images, suggesting the integrating of aptamer AS1411 and siRNA. As depicted in Fig. 2, hydrodynamic size of bare GS, Apt-GS, and Apt-GS/siRNA NGs (162.3 nm, 216.5 nm, and 234.9 nm, respectively) gradually increased along with the functionalization procedure due to an increase in the surface functional layer. On the other hand, the zeta potential of Apt-GS was less positive (33.9 ± 0.5 mV) than that of bare GS NGs (40.1 ± 0.5 mV) due to masking effect of negative DNA aptamers on the surface. After a further conjugating with siRNA on surface, the zeta potential of Apt-GS/siRNA NGs still negatively shift to 24.9 mV due to the excess-free negative phosphate groups from siRNA. This result suggested a reduced biological toxicity because the strong positive charges could interfere with negatively charged proteins in the blood and disrupt cell membrane [24].

### siRNA Loading and Release

**Fig. 3:**
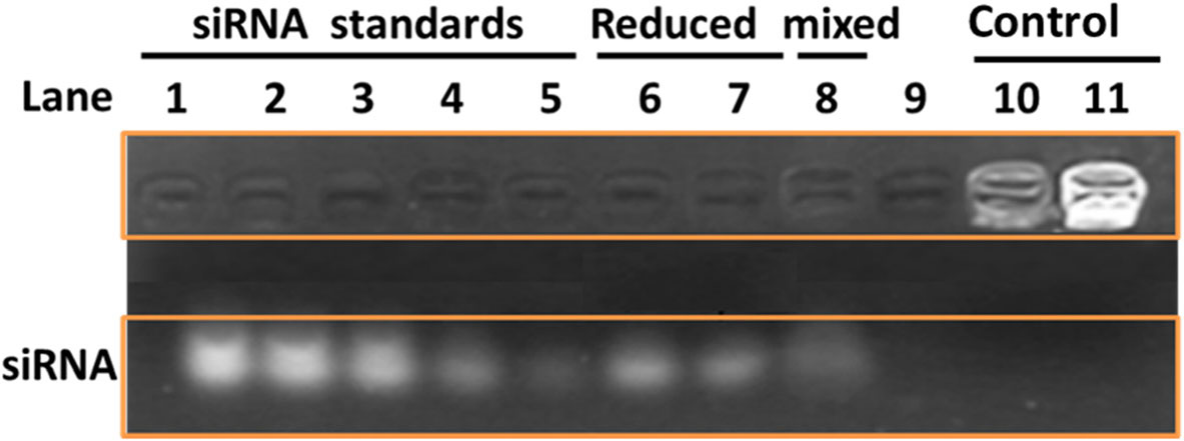
AGE analysis of siRNA reversible binding to Apt-GS nanogels. Lanes 1–5: free siRNA with amount of 50, 40, 30, 20, and 10 pmol. Lanes 6 and 7:GS/siRNA and Apt-GS/siRNA with 10 mM GSH at 2 h. Lane 8: the mixture of free siRNA and Apt-GS NGs. Lanes 10 and 11: GS/siRNA and Apt-GS/siRNA complexes without 10 mM GSH as controls

A successfully RNAi can only occur when siRNA is delivered and released rapidly from its carrier into the cytoplasm [25]. Tumor cells have 7–10 folds higher GSH concentration than that in normal cells [26, 27]. In this study, siRNA was conjugated to active Apt-GS NGs through the disulfide crosslinking reaction. The gel electrophoresis (GE) was carried out to evaluate the siRNA loading and release ability of Apt-GS/siRNA complexes. As expected, while free pDNA (Fig. 3, lanes 1–5) moved to its usual position, Apt-GS/siRNA complexes exhibited the decreased mobility in an electromobility shift assay (Fig. 3, lanes 10–11) demonstrating the good loading of siRNA. To mimick intracellular condition, 10 mM of GSH was treated with complexes for 2 h to reduce the Apt-GS/siRNA NGs. As shown in Fig. 3, lane 6–7, siRNA molecules were observed in the polyacrylamide gel after the treatment of GSH, indicating that Apt-GS/siRNA NGs could reversibly release functional siRNA molecules by disulfide cleavage in the presence of glutathione. Besides, the amount of siRNA on the nanoparticles was analyzed by comparing the total amount of siRNA and control siRNA in 2% polyacrylamide gel electrophoresis. The Apt-GS encapsulated siRNA molecules at a ratio of 3:1∼2:1 pmol per microgram. It is also noted that the mixture of siRNA and Apt-GS displayed weaker retention due to physical entrapment of negative siRNA (Fig. 3, lane 8).

### Cytotoxicity and Nucleolin Targeting

**Fig. 4:**
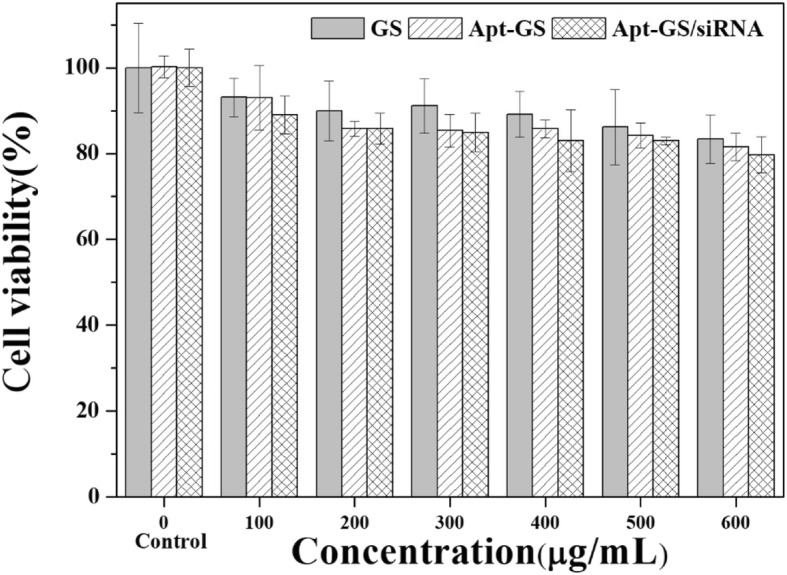
Cell viability of A549 cells with GS, Apt-GS, and Apt-GS/siRNA nanogels of various concentrations determined by MTT assay

In vitro cytotoxicity evaluation was investigated by MTT assay in A549 cells. MTT assay is used to assess the cytotoxicity of prepared nanogels. No-toxic luciferase siRNA was selected as a reporter, which has no killing effect on tumor cells [28, 29]. Hence, cell viability represented biocompatibility of carrier. As shown in Fig. 4, the cell proliferation was not remarkably affected by GS, Apt-Apt, and Apt-Apt/siRNA at the concentrations of 100–600 μg/mL groups (cell viability > 80%) and the cytotoxicity appeared dose-dependent. Compared with bare GS NGs, AS1411-modified nanogels had a slight decrease in cell viability due to the inhibition of NF-κB signaling [27, 30] and destruction of organelle membranes [16]. The benign biocompatibility of Apt-GS nanogels makes them suitable as siRNA carriers in vivo.

**Fig. 5:**
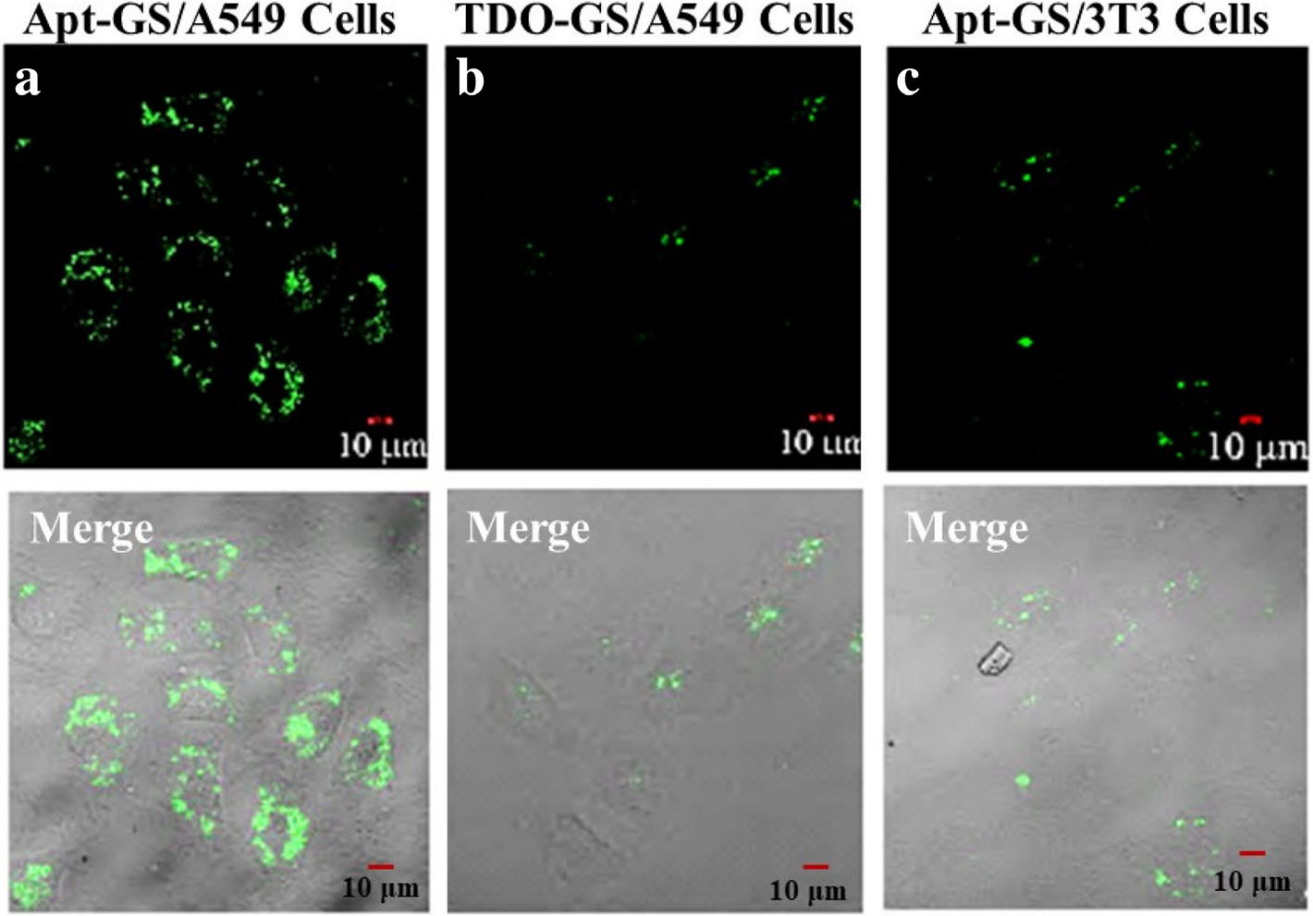
**a**–**c** Cell uptake of FAM-siRNA delivered by Apt-GS and TDO-GS nanogels. Images were taken with CLSM. Green signal: FAM. Scale bar is 10 μm

**Fig. 6:**
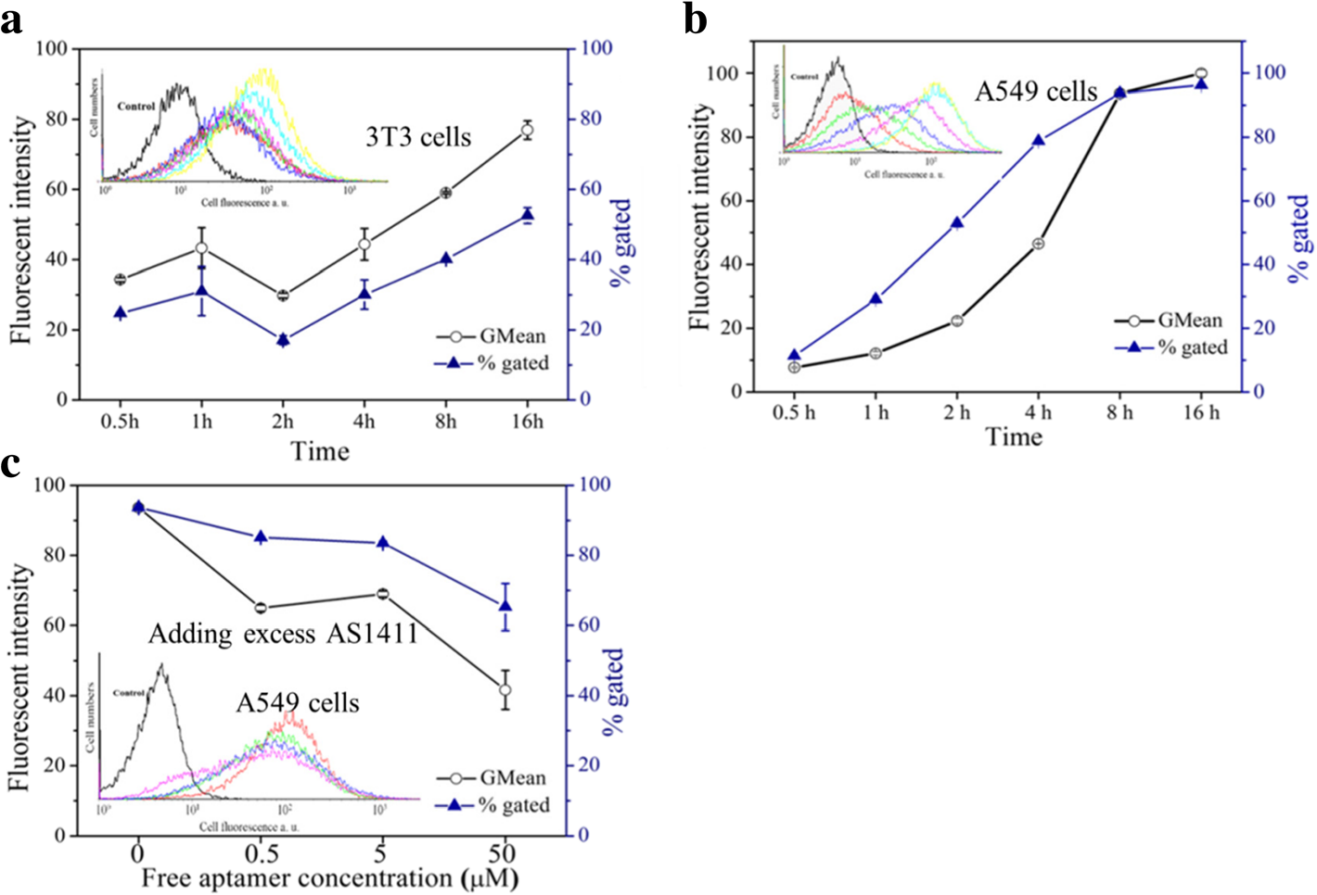
Flow cytometry analysis (FCMS) of cell uptake of FAM-labeled Apt-GS and TDO-GS nanogels in different cell types. Insert: FACS profiles, **a**, **b** curve red, green, blue, purple, aqua, and yellow present the incubation time from 0.5 to 16 h; **c** curve red, green, blue, and purple mean the concentration of AS1411 from 0 to 16 μM

To assess the targeting of Apt-GS in tumor cells in vitro, we incubated A549 cells and 3T3 fibroblasts with the same amounts of FAM-labeled Apt-GS or control TDO-GS (GS decorated with a control oligonucleotide). Low levels of fluorescence could be observed in 3T3 cells or when cells were treated with control TDO-GS (Fig. 5b, c). In contrast, green fluorescence emitted by aptamer AS1411 was markedly enhanced in A549 cancer cells (Fig. 5a), suggesting that Apt-GS was specifically accumulated in tumor cells due to the introduction of the nucleolin-targeted AS1411 aptamers. In the quantitative analysis by flow cytometry (Fig. 6), Apt-GS NGs exhibited time-dependent fluorescence intensities in both cell types. From 0.5 to 16 h fluorescence intensity of internalization of Apt-GS in A549 cells and 3T3 cells improved 13 and 2 times, respectively. As for cell ratio containing NGs, it rapidly increased in the  first 30 min and apparently maximal of possible saturation at 8 h in A549.96% of A549 cells had been attached or taken the nanogels within 16 h, while only 52% of 3T3 cells was observed. In order to evaluate the role of nucleolin in the cellular uptake of nanogels, the mixture of the prepared Apt-GS NGs and free AS1411 aptamers of varied concentrations (0.5, 5, and 50 μM) was incubated with A549 cells for 8 h. The fluorescence intensity and percentages of cells containing nanogels decreased by 50% and 30%, respectively, when using the free AS1411 aptamer to compete with the Apt-GS NGs to nucleolin expressed at the cell membrane, suggesting nucleolin-induced receptor-mediated endocytosis (Fig. 6c). These data demonstrated that Apt-GS NGs exhibited a higher accumulation in A549 cancer cells via nucleolin-mediated internalization.

### RNA Interference

**Fig. 7:**
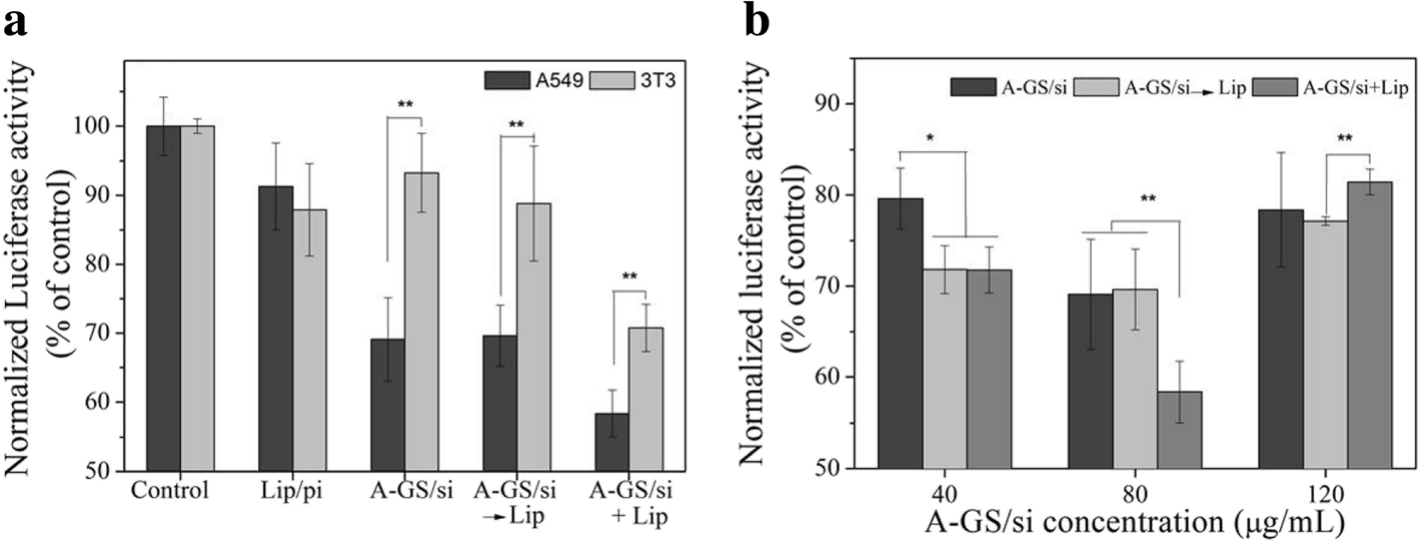
In vitro silencing effect of anti-luciferase siRNA formulated in Apt-GS nanogels with different treatments (**a**) and concentration of Apt-GS/siRNA complexes (A-GS/si) on gene silencing (**b**)

It is reported that incorporation deoxynucleotides into siRNA can increase the stability of sense strand and keep ∼ 40% efficacy of RNAi [2, 3]. To evaluate the knockdown efficiency of test siRNA carriers, we employed a ribonucleotide-replaced luciferase reporter gene system in both cells. The gene knockdown (KD) efficiency of siRNA was assessed through luciferase activity after 48 h. As shown in Fig. 7a, the Apt-GS/siRNA improves 4.6-fold gene knockdown efficiency of A549 cells compared with 6% of normal 3T3 cells, showing good cell selectivity. This suggested that the aptamer AS1411 modification enhanced transfection efficiency by generating a targeting effect. Cationic liposomes have been proven to assist cargo delivery and escape from lysosomes [31]. Moreover, the subsequent addition of liposome (A/si-GS →Lip group) did not help to improve siRNA-mediated gene silencing activity (30.4%) despite of endosome disruption, inferring siRNA release in glutathione (GSH) intracellular environment by disulfide cleavage. Besides, co-transfection of liposomes and Apt-GS/siRNA (A/si-GS + Lip group) improved slightly knockdown efficiency of luciferase expression (41.6 %) in A549 though liposome-aid lysosome escape, in correspond to previous reports [27, 30]. However, A549 cells had 2.7-fold and1.4-fold gene silencing activities for A/si-GS→Lip group and for A/si-GS+Lip, respectively, compared with 3T3 cells, inferring a decrease in cell selectivity.

Next, we examined the concentration effect of nanogels on the RNAi. As shown in Fig. 7b, gene interference efficiency reached the maximum at the complex concentration of 80 μg/ml. The decreased KD efficiency at a concentration of 120 μg/ml may due to an increased cytotoxicity from aptamer. It is also noted that addition of an endosome escape agent liposome can increase RNA interference efficiency at low concentrations of Apt-GS/siRNA nanogels (40 μg/ml and 80 μg/ml) but did not lead to a reduction at high concentrations (120 μg/ml). Thus, it is believed that Apt-GS can successfully deliver siRNA to the tumor cells and subsequently result in specific RNAi.

## Conclusions

The stability and tissue-specific delivery of siRNA remain as the biggest obstacles for RNAi therapeutics. Here, deoxynucleotide-substituted siRNA is used to improve stability of siRNA, and GS core was further coated with AS1411 aptamer for tumor targeting and disulfide linker for redox-responsive delivery of siRNA. The vehicle has a spherical structure with a size of about 200 nm and possesses positive charges at the surface. These as-prepared Apt-GS/siRNA nanogels could protect the cargo and exhibited accelerated siRNA release under DTT adding by disulfide cleavage. Moreover, with the targeting aptamer AS1411, the new Apt-GS nanogels Apt-GS could effectively deliver and release more cargo to the cytoplasm, leading to a significant (~ 5 folds in vitro improvement in silence activity in nucleolin-overexpressing A549 cells). Overall, these findings suggest that delivery of deoxynucleotide-substituted siRNA is an innovative strategy and these redox-responsive, tumor-targeting smart nanogels hold great promise for siRNA delivery and tumor therapy.

## Data Availability

The datasets supporting the conclusions of this article are included within the article.

## Abbreviations

APTMS: 3-Aminopropyl-trimethoxysilane
DMEM: Dulbecco’s modified Eagle’s medium
DMSO: Dimethyl sulfoxide
FAM: Fluorescein amidite
GE: Gel electrophoresis
GPSM: 3-Glycidoxypropyl-trimethoxysilane
GS NGs: Siloxane-crosslinked gelatin nanogels
MTT: 3-(4,5-Dimethylthiazolyl-2)-2,5-diphenyltetrazolium bromide
RNAi: RNA interference
siRNAs: Small interfering RNAs
SPDP: *N*-succinimidyl 3-(2-pyridyldithio)-propionate
TDO: Negative DNA oligonucleotide
